# Identification of Charred Cadavers by X-Rays

**Published:** 1912-10

**Authors:** 


IDENTIFICATION OF CHARRED CADAVERS BY X-RAYS. Branly, *Acad. des Sciences,* Oct. 9, 1911, commenting on a report of Fouveau of Corrmelles, in 1897, brings up the possibility of identifying corpses with destruction of features by this means, implying that for workers in extra-hazardous occupations, preliminary examinations might be made as to fractures, exostoses, irregular shadows due to variations in the mineral deposits in bone, etc.

